# Genome-wide identification and phylogenetic and expression pattern analyses of EPF/EPFL family genes in the Rye (*Secale cereale* L.)

**DOI:** 10.1186/s12864-024-10425-9

**Published:** 2024-05-30

**Authors:** Lin Zhiling, Du Wenhua, Zhao Fangyuan

**Affiliations:** College of Grassland Science, Key Laboratory of Grassland Ecosystem (Ministry of Education), Pratacultural Engineering Laboratory of Gansu Province, Sino-U.S. Centers for Grazing Land Ecosystem Sustainability, Gansu Agricultural University, Lanzhou, China

**Keywords:** Rye, EPF/EPFL gene family, Cis-element, Abiotic stress

## Abstract

Rye (*Secale cereale* L.) is one of the major cereal crop species in the Triticeae family and is known to be most tolerant to diverse abiotic stresses, such as cold, heat, osmotic, and salt stress. The EPIDERMAL PATTERNING FACTOR (EPF) and EPF-LIKE (EPFL) families of small secreted peptides act to regulate many aspects of plant growth and development; however, their functions are not widely characterized in rye. In this study, we identified 12 *ScEPF/EPFL* genes, which can be divided into six groups and are evenly distributed on six rye chromosomes. Further examination of the gene structure and protein conservation motifs of EPF/EPFL family members demonstrated the high conservation of the ScEPF/EPFL sequence. Interactions between ScEPF/EPFL proteins and promoters containing hormone- and stress-responsive cis-acting elements suggest that the regulation of *ScEPF/EPFL* expression is complex. Expression profiling analyses revealed that *ScEPF/EPFL* genes exhibited tissue-specific expression patterns. Notably, *ScEPFL1*,*ScEPFL7*, *ScEPFL9*, and *ScEPFL10* displayed significantly higher expression levels in spikelets compared to other tissues. Moreover, fluorescence quantification experiments demonstrated that these genes exhibited distinct expression patterns in response to various stress conditions, suggesting that each gene plays a unique role in stress signaling pathways. Our research findings provide a solid basis for further investigation into the functions of *ScEPF/EPFLs*. Furthermore, these genes can serve as potential candidates for breeding stress-resistant rye varieties and improving production yields.

**Supplementary Information:**

The online version contains supplementary material available at 10.1186/s12864-024-10425-9.

## Introduction

Rye (*Secale cereale.* L, 2n = 2x = 14, genome RR), a member of the Triticeae tribe of the Poaceae, is widely recognized as a significant crop for bread making and feed forage, with a particular focus on central and northeastern Europe [1]. It is valued for its high carbohydrate content and contains quantity of protein, potassium, B vitamins, lignans, ferulic acid, alkylpolysechenol, and prebiotics [2]. Notably, rye plants exhibit vigorous growth, exceptional tolerance to both abiotic and biotic stresses, and the ability to thrive in unfavorable soil conditions and cold climates [3]. Recent efforts have been made to investigate the rye genome, which has established a foundation for advancements in breeding and targeted gene editing in rye [4].

Signaling peptides serve diverse functions in intercellular communication in plants and other organisms [5, 6]. The identification of systemin as a defense signal in tomato (*Solanum lycopersicum*) [7, 8] led to the discovery of more than 15 peptide families that influence various aspects of plant development [9–11]. Among these, EPIDERMAL PATTERNING FACTORs (EPFs) and related EPF-LIKE (EPFLs) peptides are characterized by multiple conserved cysteine residues, which contribute to their structural and functional properties [12, 13]. A typical EPF/EPFL protein is marked by a signal peptide sequence at its N-terminus and 6–8 relatively conserved cysteine residues at its C-terminus, with intramolecular disulfide bond formation confirmed by Ohki et al. [14]. EPF/EPFL peptides play a crucial role in the regulation of stomatal development, with several members involved in the control of stomatal density and patterning in the plant epidermis [13].

Stomatal development is essential for plant resistance against drought, heat, and other stresses, and the modification of stomatal traits has the potential to enhance photosynthesis and improve water use efficiency to some extent [15, 16]. The distribution of stomata on the surface of leaves is a highly controlled and plastic process, and elements controlling stomatal growth are still being discovered. Multiple *EPF/EPFL* family members have been identified as regulators of stomatal development in Arabidopsis (*Arabidopsis thaliana*) [17–19] and other plant species [20–22]. In the model plant *A. thaliana*, *AtEPF1* and *AtEPF2* have negative effects on leaf stomatal development, and *AtEPFL9*/*Stomagen* is a positive regulator of leaf stomatal density and has antagonistic effects on *AtEPF1* and *AtEPF2*. In the realm of wheat, the overexpression of the *TaEPF1B* gene, as demonstrated by Dunn et al. [21], led to a reduction in stomatal density and an increase in water use efficiency. This observation suggests that the overexpression of *TaEPF1B* gene can attenuate stomatal density during the early stages of plant development, thereby sustaining photosynthesis while curtailing transpiration and water loss, consequently bolstering resilience in the face of drought and augmenting water use efficiency. Moreover, the *EPF/EPFL* genes also impact various other processes, such as filament elongation, fertility [23], stamen identity [12] and grain size in specific plant species, as observed in the case of the knockout of *OsEPFL2* in rice (*Oryza sativa*) utilizing CRISPR/Cas9 technology, leading to a shortened or awnless phenotype accompanied by reduced grain size [22]. Additionally, a plethora of studies have underscored the critical regulatory roles of the *EPF/EPFL* family in diverse physiological facets of growth and development evident in various plants, encompassing stress responses and plant phase transitions [24–28]. Despite these findings, the understanding of the *EPF/EPF*L gene family in rye has not been fully elucidated.

In this study, we utilized the recently published rye genome [4]to identify 12 *EPF/EPFL* genes, followed by a comprehensive analysis of their gene structure, motif composition, chromosomal location, and gene duplication. Furthermore, we compared the *EPF/EPFL* genes in rye with those in closely related genera to investigate evolutionary distances and relationships, with the aim of elucidating developmental mechanisms across species. Finally, we employed qRT‒PCR to analyze the expression of *EPF/EPFL* genes under stress treatment, revealing distinct expression patterns in different tissues, which initially confirmed the biological functions of *EPF/EPFL* genes in rye. The identification and analysis of *EPF/EPFL* family genes in rye provide valuable insights into the role they play in stomatal development, offering a reference for improving the drought tolerance and water-saving capabilities of rye. Additionally, this study lays the groundwork for the functional analysis of the *EPF/EPF*L gene family in other species.

## Materials and methods

### Identification and characterization of the *ScEPF/EPFL* genes

The nucleotide sequences, protein sequences, and gene annotations of the rye genome were downloaded from NCBI (https://www.ncbi.nlm.nih.gov/assembly/organism/4550/latest/). The rye genome was scanned using HMMER software based on HMM profiles of EPF/EPFLs obtained from the Pfam database (pfam17181) (https://www.ebi.ac.uk/interpro/entry/pfam/PF17181/, accessed on 26 March 2023), after which the retrieved protein sequences were searched for the EPF/EPFL domain in the CDD database (https://www.ncbi.nlm.nih.gov/cdd/?term=, accessed on 28 March 2023) and SMART database (http://smart.embl.de/, accessed on 28 March 2023). The non-EPF/EPFL domain genes were removed to obtain the final list of ScEPF/EPFL genes. Based on the annotations, these genes were mapped onto chromosomes, and a structural map was constructed using MapChart [29]. The sequence manipulation suite was used to determine the molecular weights and isoelectric points of the identified proteins [30].

### Sequence and phylogenetic analysis

The full-length EPF/EPFL amino acid sequences of EPF/EPFL in rye were aligned using MEGA 7.0 [31]. Conserved motifs for the predicted ScEPF/EPFL protein sequences were identified using the MEME online program7 with default settings, except that the motif number was set to 10 [32]. Gene structure and motif distribution were visualized using TBtools software [33]. The gene sequences and annotation files of *Arabidopsis thaliana* (At), *Oryza sativa* (Os) and *Triticum aestivum* (Ta) were downloaded from the Ensembl database (https://plants.ensembl.org/index.html, accessed on 20 May 2023) and subsequently used to construct a phylogenetic tree (Table S5). MEGA 7.0 was used to construct phylogenetic trees via the neighbor‒joining method with the Poisson model, pairwise deletion, and 1,000 bootstrap replications [31]. The cis-acting elements in the 2,000 bp upstream sequence of the ScEPF/EPFL promoter were predicted using PlantCARE (http://bioinformatics.psb.ugent.be/webtools/plantcare/html/, accessed on 25 May 2023), and TBtools was used to visualize the cis-acting elements [34].

### Chromosome distribution, gene duplication, and collinearity analysis

The chromosomal location of the rye *EPF/EPFL* gene was obtained from the genome assembly files, and the chromosomal distribution was mapped using TBtools. Collinearity analysis of the 12 *ScEPF/EPFL* genes was performed using TBtools software to detect gene duplication events. Based on the results of the collinearity analysis, nonsynonymous (Ka) and synonymous (Ks) substitutions were calculated for each pair of duplicated genes using TBtools. The Ka/Ks ratio was used to analyze selection pressure (Table S2). Finally, the homology of the *EPF/EPFL* genes between rye and *T. aestivum* was measured using Dual Synteny Plotter (https://github.com/CJ-Chen/TBtools, accessed on 5 October 2023). The three-dimensional structures of the EPF/EPFL peptides were modeled with SWISS-MODEL software (SWISS-MODEL (expasy.org), accessed on 19 November 2023).

### Plant materials and stress treatments

The seeds of “Gannong 2” rye (Grassland College of Gansu Agricultural University) were germinated on germination paper (Anchor paper Co., MN, USA) at 25 °C for one week. Seedlings exhibiting synchronized growth were transferred to a hydroponic box containing one-half Hoagland’s solution and allowed to float using a hydroponic sponge. The plants were grown in a controlled environment with a 16/8 h photoperiod and 25 °C temperature. When the fourth leaf appeared, abiotic stress treatments were conducted as described hereafter. To simulate drought stress, the plants were grown in Hoagland’s solution containing 30% polyethylene glycol (PEG 6000). To determine the effect of heat and cold stress on gene expression over time, plants were sampled at 0, 1, 3, 6, 12, and 48 h after the 37 °C treatment, and 7 days after the 4 °C treatment, with three replicates per treatment.

### Gene expression analysis

RNA-seq analysis was performed to observe rye tissue-specific gene expression in the ScEPF/EPFLs. The RNA sequencing data of rye roots, stems, leaves, spikes, and maturing seeds were downloaded from the NCBI SRA (accession no. PRJNA680499). Sequencing reads were mapped to the reference genome of the rye (The IRGSC, 2020) using the HISAT2 program [35]. The number of mapped reads on each ScEPF/EPFL gene was counted using feature counts [36], and the differential gene expression among the different rye tissues was represented as a heatmap constructed using TBtools software.

Total RNA was extracted from each plant sample using the Tiangen Total RNA Extraction Kit (Tiangen Biochemical Technology Co., Ltd., China), and first-strand cDNA was synthesized from 2 µg of total RNA using HiScript III R RT SuperMix (+ gDNA wiper) for qRT‒PCR (Vazyme Biotech Co., Ltd., Nanjing, China). First-strand cDNA was synthesized from 2 µg of total RNA using Primer 6. The sequences of the primers used for qRT‒PCR were designed using Primer 6, and the sequences of the primers used for qRT‒PCR are shown in Table S4. ScActin (GenBank Accession: FJ032189.1), known for its constant expression, was used as the internal control gene. Real-time PCR was performed using EvaGreen 2X qPCR MasterMix (Sevier Biotechnology Co., Ltd., China) and a LightCycler-96 real-time PCR instrument (Bio-Roche, Switzerland). Three independent replicates were performed. The Ct values obtained for each gene were normalized to the internal gene control, and the gene expression levels were calculated using the 2^−ΔΔCT^ method [37].

## Results

### Identification of EPF/EPFL genes in ryes

Based on the Hidden Markov Model (HMM) profile of the conserved EPF/EPFL domain (pfam17181), a genome-wide scan was performed to identify the EPF/EPFL gene family of rye. After removing the redundant sequences, 13 *EPF/EPFL* candidate genes were identified. The protein sequences of these candidate genes were re-examined utilizing the NCBI CDD and Pfam databases to remove genes without the EPF/EPFL domain. Finally, 12 *ScEPF/EPFL* genes were obtained. The genes were named *ScEPF1*- *ScEPF2* and *ScEPFL1*- *ScEPFL10 according to their chromosomal position* (Table 1). The *ScEPF/EPFL* proteins examined in this study varied in length from 63 amino acids (*ScEPFL5*) to 146 amino acids (*ScEPF1/ScEPFL2*). Their molecular weights ranged from 7144.31 Da (*ScEPFL5*) to 16,158.86 Da (*ScEPFL2*), and their isoelectric points ranged from 7.54 (*ScEPF2*) to 10.2 (*ScEPFL6*) (Table 1).

**Fig. 1:**
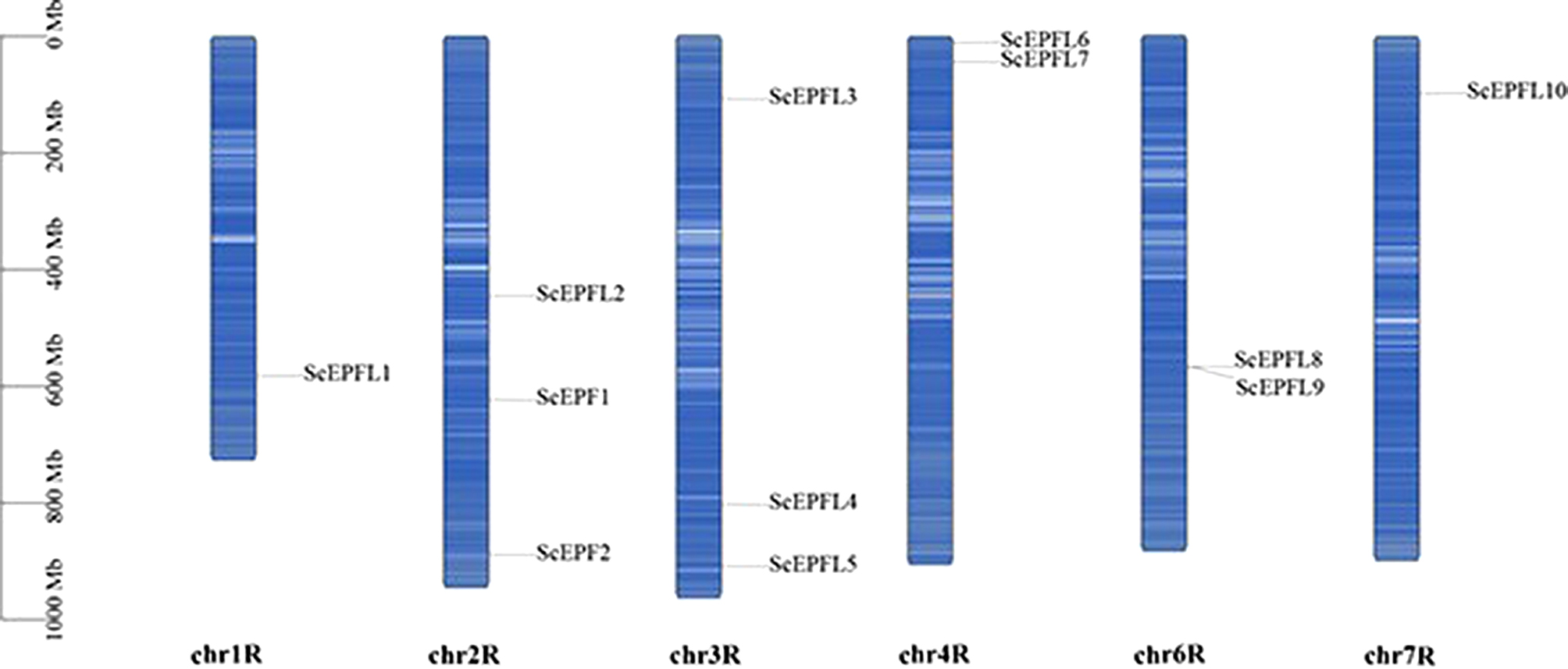
The distribution of *ScEPF/EPFLs* on rye chromosomes. The scale represents the length of the chromosomes, whereas the blue bars represent each chromosome, and the black lines indicate the position of each *ScEPF/EPFL* gene

Mapping revealed an uneven distribution of the *ScEPF/EPFL* genes on the six rye chromosomes (Fig. 1), and these genes were not detected on the other chromosomes of the rye. The eye *EPF/EPFL* genes were found on chromosomes 1–7, except for chromosome 5. Only one *ScEPF/EPFL* gene was found on chr1 and chr 2. Three *ScEPF/EPFL* genes were found on chromosomes 2 and 3, respectively.

**Table 1:** The *EPF/EPFL* gene family in rye

Name	Gene ID	Chr	Position	Length (aa)	MW (Da)	pI
ScEPF1	SECCE2Rv1G0105600	2	623,016,917–623,017,483	146	15298.36	8.94
ScEPF2	SECCE2Rv1G0132300	2	889,033,470–889,033,957	134	14070.35	7.54
ScEPFL1	SECCE1Rv1G0042640	1	582,536,439–582,537,660	128	13396.61	9.21
ScEPFL2	SECCE2Rv1G0096780	2	444,761,952–444,762,584	146	16158.86	7.59
ScEPFL3	SECCE3Rv1G0160890	3	109,525,293–109,525,739	118	12788.8	9.65
ScEPFL4	SECCE3Rv1G0195960	3	804,275,330–804,276,616	144	15607.09	10.15
ScEPFL5	SECCE3Rv1G0206730	3	910,611,950–910,612,810	63	7144.31	9.19
ScEPFL6	SECCE4Rv1G0216540	4	11,366,349–11,366,811	123	13130.26	10.2
ScEPFL7	SECCE4Rv1G0221020	4	44,073,984–44,074,448	123	13290.43	9.54
ScEPFL8	SECCE6Rv1G0408100	6	568,321,805–568,322,272	121	12493.26	9.63
ScEPFL9	SECCE6Rv1G0408210	6	569,287,410–569,287,877	121	12580.33	9.63
ScEPFL10	SECCE7Rv1G0468440	7	98,292,518–98,292,965	113	12213.11	9.2

Chr: chromosome, aa: amino acid, MW: molecular weight, pI: isoelectric point

### Phylogenetic analysis of the *ScEPF/EPFLs*

**Fig. 2:**
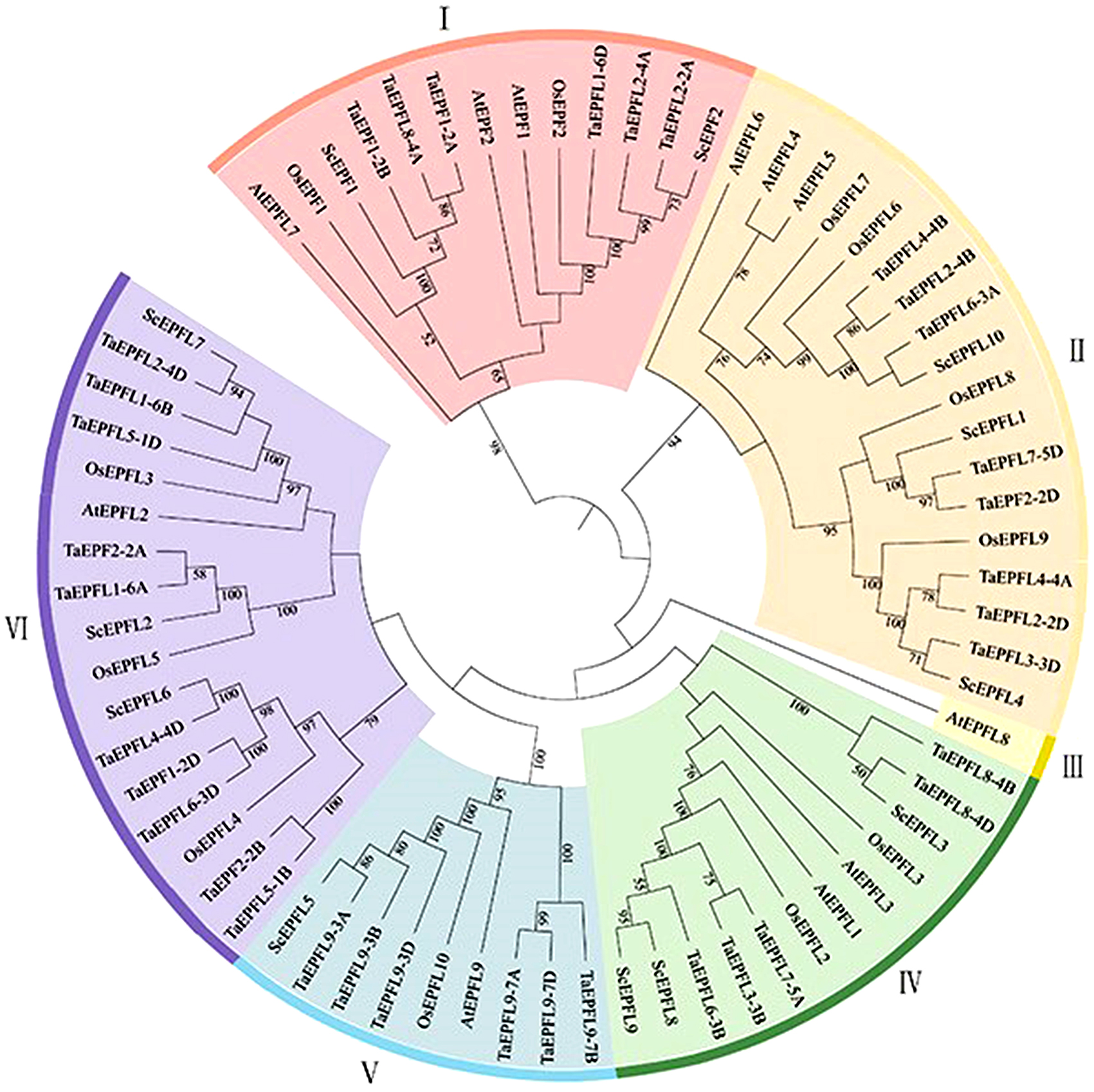
Phylogenetic analysis of the *EPF/EPFL* genes of *Secale cereale* (Sc), *Triticum aestivum* (Ta), *Oryza sativa* (Os), and *Arabidopsis thaliana* (At). MEGA 7.0 software was used to construct a neighbor-joining phylogenetic tree with 1,000 bootstrap replications. Subgroups are highlighted with different colors

To clearly demonstrate the evolutionary relationships of the strains, we constructed a phylogenetic tree with protein sequences of 12 *EPF/EPFLs* from rye, 12 *EPF/EPFLs* from rice, 35 *EPF/EPFLs* from wheat and 11 *EPF/EPFLs* from Arabidopsis via MEGA 64 via the neighbor-joining (NJ) method. Seventy EPFs were divided into six subgroups (I–VI) (Fig. 2). Subgroups I-II and IV-VI contain 13, 18, 12, 10 and 16 EPF/EPFL proteins, respectively, whereas subgroup III contains only one gene, AtEPFL8. 12 EPFs/EPFLs are classified in subgroups I, except for III: There are more *ScEPF/EPFLs* in subfamilies II and IV (three *ScEPF/EPFLs*, respectively) and two *ScEPF/EPFLs* in subfamilies I, V, and VI. Functional clustering. A comparison with the phylogenetic tree of rye revealed that most of the *ScEPF/EPFLs* were closely clustered with *TaEPF/EPFL*, which could explain the close proximity between rye and wheat, suggesting that these proteins may be homologous and have similar biological functions in both rye and wheat.

### Sequence and structural analysis of the *ScEPF/EPFLs*

**Fig. 3:**
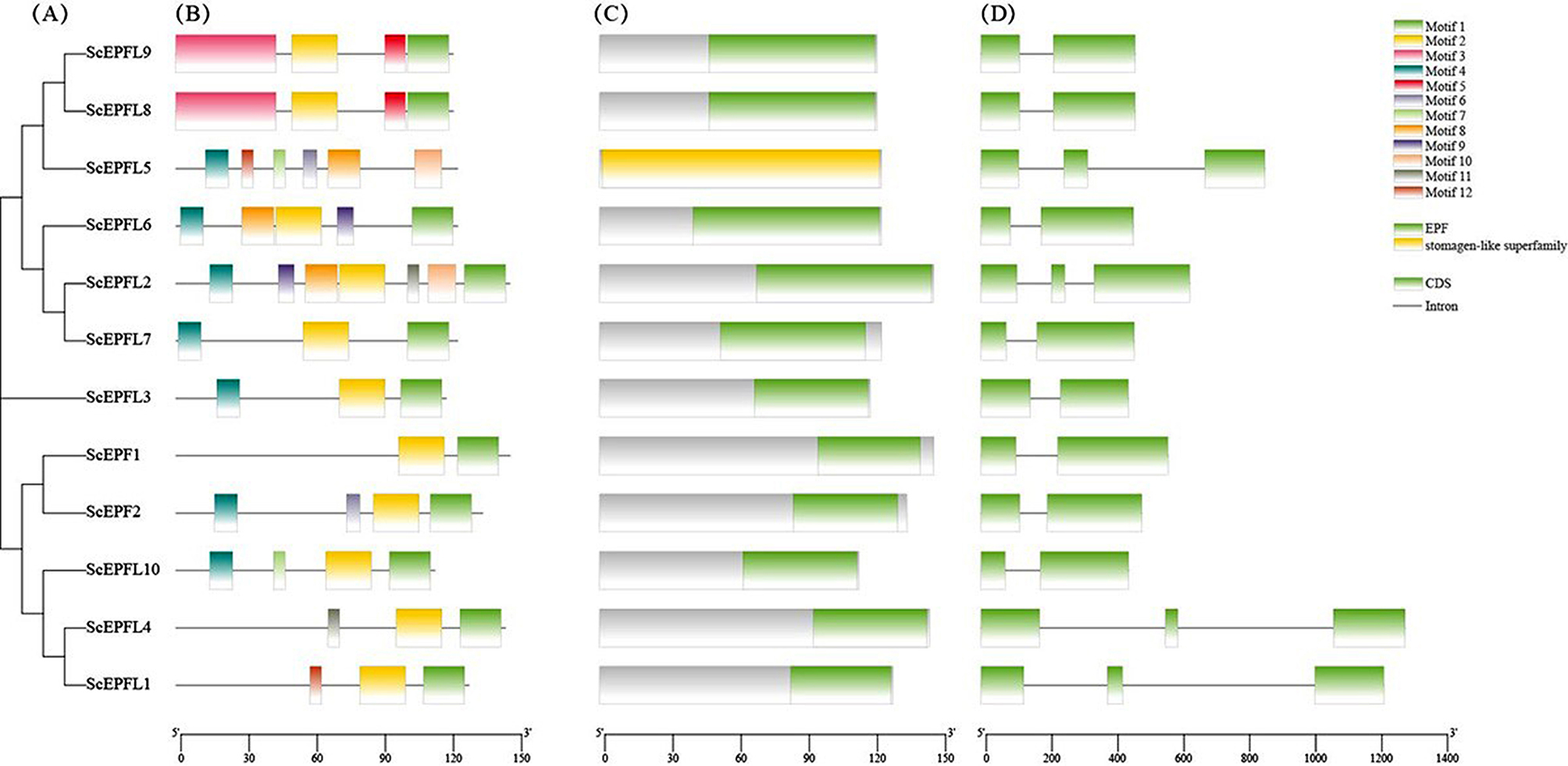
Analysis of the phylogenetic relationships, motifs, and gene structures of growth-regulating factor genes from *S. cereale* L. (A) Phylogenetic tree of 12 *ScEPF/EPFLs* in rye. (B) Conserved motif arrangements of the *ScEPF/EPFLs*. The 12 different colored boxes represent the different structural domains and their positions in each *EPF/EPFL*. (C) Conserved protein structural domain family of *ScEPF/EPFLs*. The green boxes indicate EPF families; the yellow boxes indicate stomagen-like families. (D) Exon intron organization of the *ScEPF/EPFLs*. The green boxes indicate exons; the gray lines indicate introns

The gene structure and motif characteristics of the genes were analyzed (Fig. 3A–D). A total of ten motifs were identified from these *ScEPF/EPFLs*. The multilevel consensus sequences for the MEME-defined motifs are shown in Table S1. All the *ScEPF/EPFLs* contained different numbers of motifs, ranging from 2 to 7. All the *ScEPF/EPFL proteins* had motif 1 and motif 2, except for *ScEPFL5*, whose motifs 1 and 2 were located close to each other. Two members of subgroup (*ScEPFL8* and *ScEPFL9*) contained four identical motifs (Figs. 2 and 3B). *ScEPFL1* contained the lowest number of motifs. *ScEPFL5* belongs to the stomagen-like superfamily, and the other 11 *ScEPF/EPFLs* are all EPF gene families (Fig. 3C).

We investigated the exon–intron organization of the *ScEPF/EPFLs* and constructed schematic diagrams of their protein structures to better understand their evolutionary relationships and functions. The *ScEPFL1, ScEPFL2, ScEPFL4, and ScEPFL5* genes had three exons and two introns, and the other *ScEPF/*EPFL genes had two exons and one intron. The members of each subgroup in the phylogenetic tree were similar in size and contained similar genetic structures (Figs. 2 and 3D), indicating their conserved functions. The secondary structures of the *ScEPF/EPFLs* were mainly random coils (39.55–66.12%) and alpha helices (0.31–35.40%), while the extended strand accounted for 4.13–19.51%, and the beta turn accounted for 3.31–13.19% (Table 2). Furthermore, we projected the three-dimensional structure of the *EPF/EPFL*s in the Secale cereale. All the EPF/EPFL domains in the *ScEPF/EPFLs* had random coil and alpha helix topologies according to the results, with certain *EPF/EPFL* domains in the *ScEPF/EPFLs* having beta turns (Fig. S1). These findings demonstrated patterns of diversification and conservation among the *ScEPF/EPFL* proteins during the course of evolution.

**Table 2:** Secondary structure prediction of the *ScEPF/EPFL* proteins

Protein	Alpha helix (Hh)	Extended strand (Ee)	Beta turn (Tt)	Random coil (Cc)
ScEPF1	25.34%	10.27%	5.48%	58.90%
ScEPF2	9.55%	10.45%	10.45%	39.55%
ScEPFL1	0.31%	17.19%	10.94%	51.56%
ScEPFL2	29.45%	10.96%	3.42%	56.16%
ScEPFL3	16.95%	19.49%	9.32%	54.24%
ScEPFL4	20.14%	15.28%	13.19%	51.39%
ScEPFL5	21.95%	19.51%	8.13%	50.41%
ScEPFL6	26.02%	9.76%	4.88%	59.35%
ScEPFL7	3.58%	13.01%	4.88%	58.54%
ScEPFL8	26.45%	4.13%	3.31%	66.12%
ScEPFL9	29.45%	10.96%	3.42%	56.16%
ScEPFL10	35.40%	4.42%	6.19%	53.98%

### Gene duplication and collinearity analysis

**Fig. 4:**
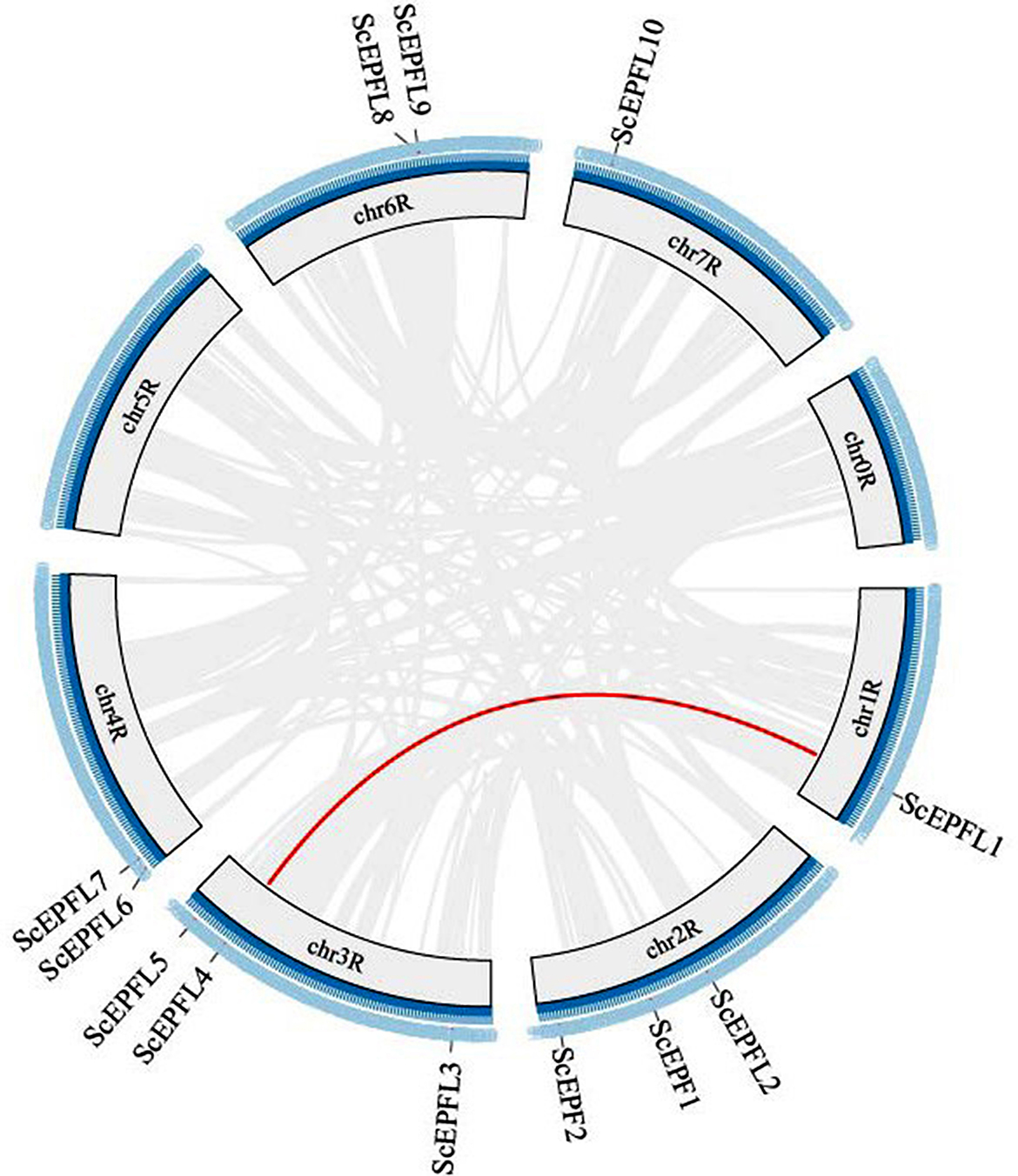
The synteny blocks of *EPF/EPFL* genes in rye. Analysis of interchromosomal fragment duplication of *EPF/EPFL* genes in the rye genome. The gray lines represent all synthetic blocks, and the red lines specify the duplicated pairs among the 12 *ScEPF/EPFL* genes

A duplication event analysis of the *EPF/EPFL* genes in rye showed that there were no tandem duplication events in the rye genome, but one pair of duplicated fragments was present (Fig. 4). For every pair of duplicated genes, the synonymous substitution rate (Ks) and nonsynonymous substitution rate (Ka) were determined (Table S2). All the gene pairings had Ka/Ks values less than 1, suggesting that purifying selection was at work on the *ScEPF/EPFL* gene family.

**Fig. 5:**
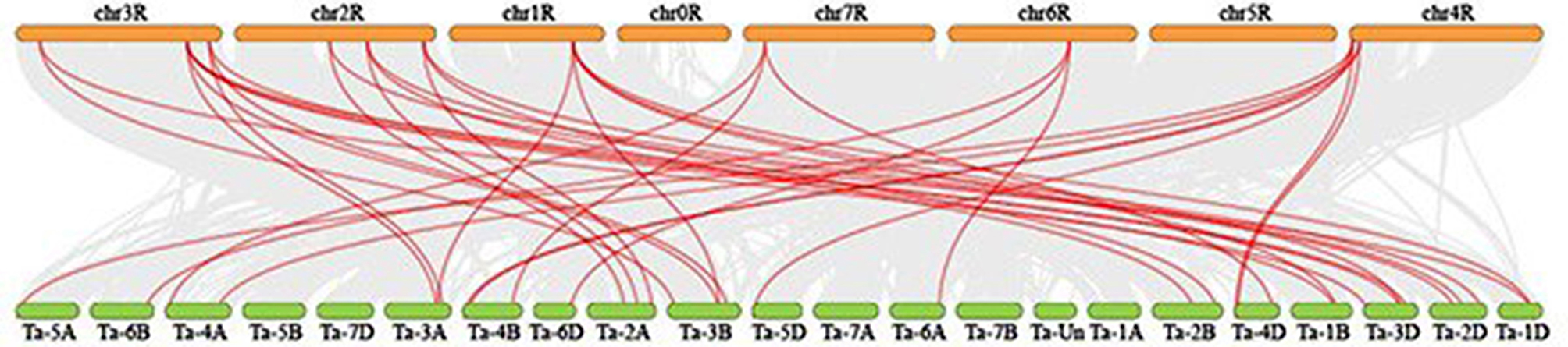
Analysis of the *EPF/EPFL* genes between *rye* and *T. aestivum*. The gray lines in the background indicate the neighboring blocks in the genomes of *rye* and *T. aestivum*, whereas the red lines highlight the syntenic rye *EPF/EPFL* gene pairs

Homology analysis of these two plants revealed at least one pair of genes homologous to *ScEPF/EPFL*, and each *ScEPF/EPFL* gene had 2–3 wheat *EPF/EPFL* genes homologous to it, indicating that these homologous genes were highly conserved and might have existed prior to ancestral divergence (Fig. 5). Accordingly, it was speculated that these genes might have played a crucial role in the evolution of the *EPF/EPFL* gene family in rye.

### Cis-acting element analysis of the *ScEPF/EPFLs*

**Fig. 6:**
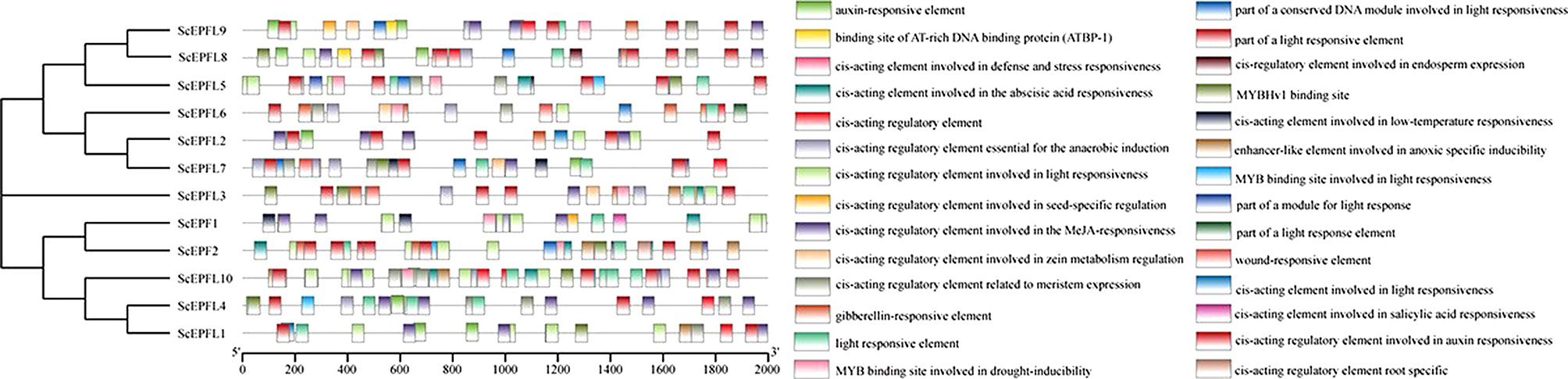
The cis-acting element is contained within the 2 kb promoter sequence of the *ScEPF/EPFL* gene. Different cis-elements are indicated by different colored rectangles and are placed in the matching position on the promoter

The online PlantCARE cis-element database was used to analyze the promoter sequences (upstream 2,000 bp) of the *ScEPF/EPFL* genes. The conserved TATA-box and enhancement CAAT-box elements in the promoter sequences were observed to conform to the basic structural characteristics of eukaryotic gene promoters. The promoter sequence also contained many elements related to hormonal and abiotic stress responses (Fig. 6). Hormone-responsive elements include salicylic acid cis-acting elements, gibberellin-responsive elements, abscisic acid-responsive elements, and auxin-responsive elements. Abiotic stress response elements include anaerobic inducible elements, light responsive elements, low temperature responsive cis-acting elements, and MYB binding sites involved in drought inducibility. In addition, we found certain unique cis-acting elements in the promoter sequence: cis-acting regulatory elements related to meristem expression, cis-regulatory elements involved in endosperm expression, cis-acting regulatory elements involved in root specificity and cis-acting regulatory elements involved in seed-specific regulation. Each *ScEPF/EPFL* contains at least one hormone-related cis-element and one stress-related cis-acting element; however, the types of these elements vary.

### Expression analysis of *the ScEPF/EPFL* genes

**Fig. 7:**
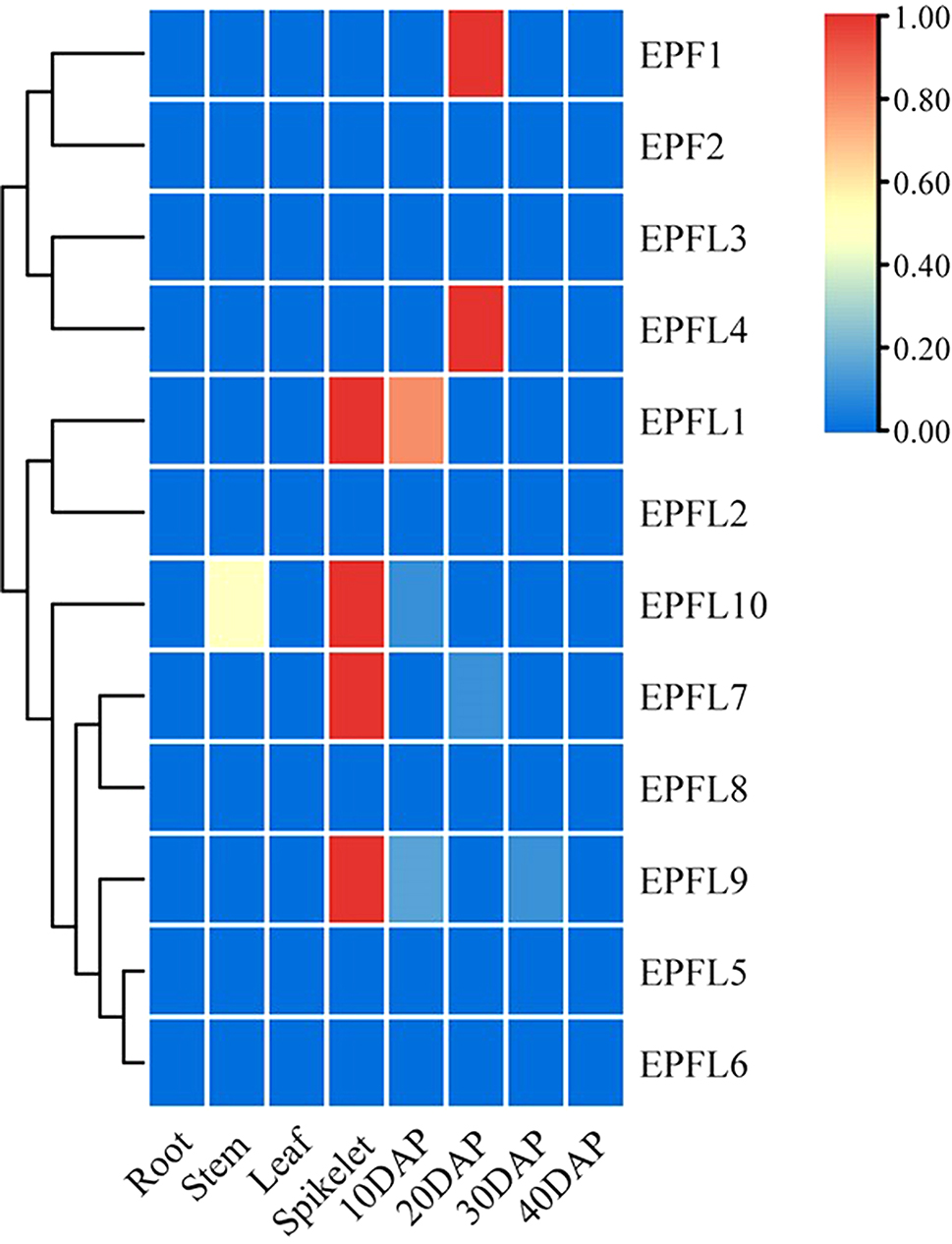
A heatmap of rye tissue-specific gene expression of the *ScEPF/EPFL* genes. The genes were clustered using the average linkage method with Euclidean distance measurements. DAP; Days after pollination

To explore the biological functions of *ScEPF/EPFL*s during growth and development, we assessed the transcript abundance patterns of *ScEPF/EPFL*-encoding genes in a total of eight different tissues, leaves, stalks, roots, spikes, and seeds at 10, 20, 30, and 40 days after pollination (DAP), based on the downloaded rye genotypic expression databases (Fig. 7). ScEPFL1, ScEPFL7, ScEPFL9 and ScEPFL10, which were highly expressed in spikes, while ScEPFL1 and ScEPFL10 were highly expressed in seeds at 10DAP and stems, respectively. The EPF1 and ScEPFL4 genes highly expressed in seeds and stems at 20DAP; ScEPF2, ScEPFL2, ScEPFL3, ScEPFL5, ScEPFL6, and ScEPFL8, which were barely expressed in the tissues.

**Fig. 8:**
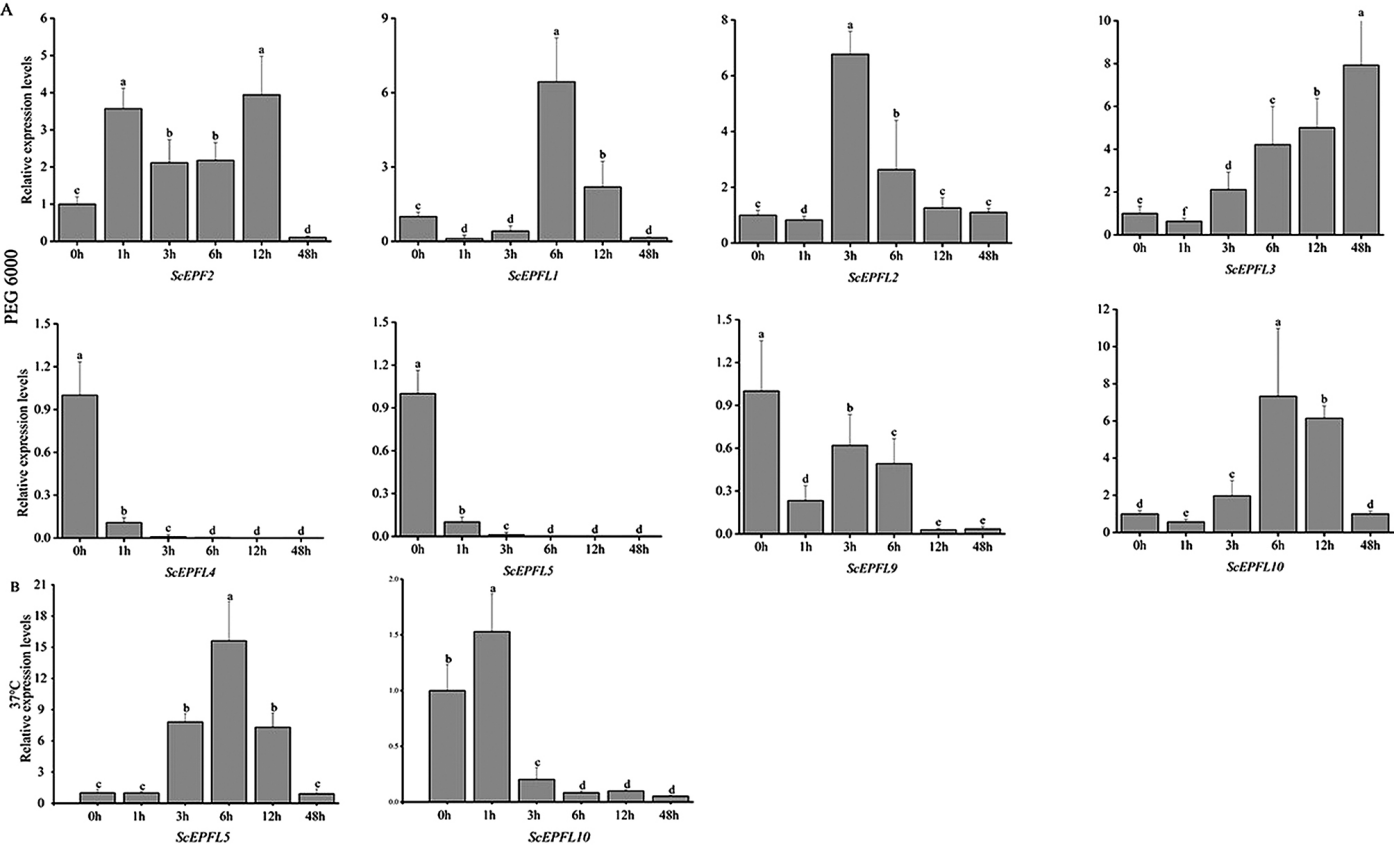
The gene expression levels of the *ScEPF/EPFL* genes under abiotic stress conditions were quantified via qRT‒PCR and are shown as bar graphs (A, B). Different letters (a, b, c, etc.) indicate significant differences at *P* < 0.05 according to Student’s t test

To analyze the expression of the *ScEPF/EPFL* genes under different abiotic stresses, rye plants were subjected to cold, heat and polyethylene glycol (PEG) treatments and observed at different time points (Fig. 8A). The expression of *ScEPFL2*, *ScEPFL3* and *ScEPFL10* increased significantly after 3 h of PEG6000 treatment, and the expression of *ScEPF2* increased abruptly at 1 h with prolonged PEG6000 treatment; however, the expression of *ScEPF2* decreased sharply after 48 h and was significantly lower than that at 0 h (*P* < 0.05). The expression of *ScEPFL4*, *ScEPFL5*, and *ScEPFL9* decreased dramatically at 1 h of PEG6000 treatment, especially *ScEPFL4* and *ScEPFL5* had minimal expression after 3 h (*P* < 0.05).

Under heat stress, the expression of *ScEPFL5* was highest at 6 h after treatment, and then decreased with the prolongation of heat stress (*P* < 0.05); its expression was not different from that of the control 0 h after 48 h of heat treatment. The expression of *ScEPFL10* surged after 1 h of heat stress, and then the expression of *ScEPFL10* decreased abruptly with the increase of heat stress, which was significantly lower than that at 0 h (*P* < 0.05, Fig. 8B).

There was no change in the expression of *ScEPF/EPFLs* after 7 days of low-temperature stress (Fig. S2).

## Discussion

EPIDERMAL PATTERNING FACTOR (EPF) and EPF-LIKE (EPFL) family genes are widely distributed as small secreted peptides in plants, which play crucial roles in plant growth, development, and stress tolerance [22]. With advances in sequencing technology, an increasing number of *EPF/EPFL* genes have been identified in a wide range of plants including poplar, Arabidopsis, tomato and rice [22, 38, 39]. Although the reference genome of rye has been published [40], a comprehensive understanding of the *EPF/EPFL* gene family in rye is still lacking.

In this study, we identified the epidermal patterning factor (EPF-like) gene family in rye by analyzing the recently published rye genome [4]. To the best of our knowledge, our work is the first report on rye *EPF/EPFL* genes. Our analysis revealed that 12 *EPF/EPFL* genes were identified in rye, which was similar to that in other species, including 11 in *Arabidopsis thaliana* [41], 12 in rice [22], and 35 in wheat [42]. The distribution of *EPF/EPFL* family members in different species indicates that *EPF/EPFL* genes play unique roles in the stress response and regulation of plant growth and development in different species. These differences suggest that the *EPF/EPFL* family genes may have evolved independently after the divergence of these species [38].

Phylogenetic analysis revealed that the 12 *ScEPF/EPFLs* could be divided into six subgroups according to their phylogenetic relationships (Fig. 2). *AtEPF1-EPF2* and AtEPFL9/Stomagen have been shown to act antagonistically in regulating leaf stomatal density [43]. However, there are already published studies showing a positive relationship between EPF/EPFL gene expression and water use efficiency (WUE) using MdEFL2 gene isolated from apple and expressed in tomato, e.g., Jiang et al. [39]. Thus, the function of *ScEPF/EPFLs* in the same subgroup can be inferred based on clustering in the evolutionary tree, which provides a foundation for future studies on the mechanism through which *EPF/EPFLs* control leaf stomatal development. The gene structure and motif distribution were consistent with the phylogenetic results, which confirmed the phylogenetic relationships among the ScEPF and EPFL genes (Fig. 3). Members of the same subgroup harbor similar gene structures and conserved motifs. Most genes in the *EPF/EPFL* family contain two introns [12, 23], as revealed in earlier studies, and our results of gene structure analysis are consistent with this observation (Fig. 2D).

Gene duplication is considered to be a primary driving force in the evolution of genomes and genetic systems [44]. Tandem duplication events and large-segment duplication events are considered the main mechanisms responsible for the expansion of gene families in the genome [45]. Based on their physical location, 12 *ScEPF/EPFL* genes were unevenly distributed on 7 chromosomes of the ryes (Fig. 1). Homology analysis of the *EPF/EPFL* gene in Rye revealed no tandem duplicate gene pairs. Nevertheless, one pair of fragment duplicates was identified (Fig. 2). When the Ka/Ks of all the replicating gene pairs was less than 1 (Supplementary Table 2), most of the nonsynonymous substitutions were harmful, indicating that the environmental selection pressure during the evolutionary process was negative and that the *ScEPF/EPFL* genes were selected for purification. Moreover, the high covariance between rye and its close relative wheat inferred that *EPF/EPFL* may have been subjected to strong evolutionary constraints during the evolutionary process, suggesting the conserved function of its family genes, which may play an important role in basic signal transduction pathways, such as the maintenance of normal physiological functions in plants.

Analysis of the promoters of the rye annexin genes revealed the presence of several cis-elements associated with the light response, indicating the potential regulation of the rye *EPF/EPFL* gene by light and its involvement in plant resilience against adverse conditions. Notably, previous experimental findings by Liu et al. [25] demonstrated that the expression of *ZmEPF2*, which is induced by blue light, was controlled by *ZmCOP1*, resulting in increased stomatal formation, as evidenced by a greater stomatal index and greater density. In our study, PlantCARE was used to predict cis-acting elements located 2,000 bp upstream of the promoter, suggesting the potential role of these elements in response to moisture stress and ABA-mediated stomatal closure. The *ScEPF/EPFL* genes encompassed abiotic stress-related cis-acting elements and light-responsive elements, indicating their responsiveness to various stresses and suggesting that they are promising candidate genes for further exploration in the context of abiotic stress research.

Most of the *ScEPF/EPFL* genes exhibited distinct patterns of tissue-specific expression during rye growth and development, with some being specifically expressed in young rye spikes and young grains where *ScEPF1* and *ScEPFL9* have cis-acting regulatory element involved in seed-specific regulation, indicating their potential roles in the early stages of reproductive growth. This observation aligns with the known functions of Arabidopsis *EPF/EPFL* genes which are involved in regulating stamen and inflorescence development and influencing stomatal density. It has been reported that *AtEPFL4* and *AtEPFL6* mainly act as positive regulators of inflorescence growth under normal conditions in *Arabidopsis thaliana*, and *ERECTA* can sense two *EPFLs* peptides to promote the growth of inflorescence stems. *ScEPFL10* and *AtEPFL4*, *AtEPFL6* are in subgroup II of the phylogenetic tree, while *ScEPFL10* was highly expressed in spikes and stems one week after anthesis, and it was inferred that *ScEPFL10* may physically bind to the receptor structural domain of *ERECTA* in rye to coordinate the proliferation of above-ground tissue layers. Notably, a few *ScEPF/EPFL* genes were not detected in different tissues, possibly due to the characteristic action of *EPF/EPFL* peptide hormones as extracellular ligands, which initiate downstream signaling cascades by binding to cell membrane surface receptor kinases [46]. These extracellular signaling factors are typically present at low concentrations and are subject to rapid, cell-specific regulation to maintain specific concentrations. Further investigation is warranted to elucidate the potential complex regulatory mechanisms through which these undetected genes may carry out their specific functions.

The *EPF/EPFL* gene family is regulated differently by different abiotic stresses, such as cold, heat and PEG, according to previous studies on various plant species. Overexpression of *HvEPF10* in barley significantly improved drought tolerance, water use efficiency, and soil water retention by reducing stomatal density [27]. Wheat plants overexpressing *TaEPF1-2B* showed lower stomatal density and higher water use efficiency to improve drought stress tolerance. *ScEPF1*,*ScEPF2* and *TaEPF1-2B* were in subgroup of the phylogenetic tree, and it was inferred that *ScEPF1* and *ScEPF2* could improve water use efficiency by inhibiting stomatal differentiation, thus improving drought stress tolerance in rye [38]. Additionally, *PagSTOMAGEN* was shown to increase water use efficiency and drought tolerance by influencing the genes associated with photosynthesis and phytohormones. This gene has been shown to enhance the nutritive growth of *Populus* by increasing the photosynthetic rate and phytohormone content (IAA, IPR) through the modulation of genes involved in photosynthesis and phytohormone pathways [47]. Ectopic overexpression of *AtEPFL9* in Arabidopsis and *OsEPFL9* in rice increased stomatal density, and *ScEPFL5*, *ScEPFL4* were in groups VI and II with the former two, respectively, inferring that the reduction of *ScEPFL5*, *ScEPFL4* was a manifestation of adaptation to drought stress in rye. It has been demonstrated that *ScEPF/EPFLs* are induced to express under abiotic stress and exhibit diverse expression levels, suggesting that *ScEPF/EPFLs* may play various roles in plant growth and development. While the aforementioned findings suggest that *ScEPF/EPFLs* are implicated in rye’s response to abiotic stress, further research is needed to understand the underlying mechanism.

## Conclusion

In summary, this study successfully identified 12 novel Sc*EPF/EPFL* gene families in rye. Sequence analysis revealed the presence of specific structural domains related to epidermal pattern factors in these genes. Phylogenetic tree analysis further classified the *EPF/EPFL* genes into six distinct categories while chromosomal localization analysis demonstrated a high level of conservation in the homologous lineage of *EPF/EPFL* genes between rye and wheat. Tissue-specific gene expression analysis indicated that *EPF/EPFLs* were primarily expressed in young tissues. Additionally, these genes exhibited differential expression patterns under various abiotic stress treatments suggesting that they play diverse roles in conferring abiotic stress tolerance. The findings presented in this study contribute to a fundamental understanding of *EPF/EPFLs* and their involvement in abiotic stress response mechanisms.

### Electronic Supplementary Material

Below is the link to the electronic supplementary material.

Supplementary Material 1

Supplementary Material 2

## Data Availability

Data is provided within the manuscript or supplementary information files. The genome assembly data and RNA-seq data utilized in this investigation were obtained from the NCBI Sequence Read Archive (SRA) database (https://www.ncbi.nlm.nih.gov/sra) under accession code PRJNA680499.
