# Supplementary material for: Genome-wide identification and phylogenetic and expression pattern analyses of EPF/EPFL family genes in the Rye (Secale cereale L.)
Source: BMC Genomics. 2024 May 30;25:532. doi: 10.1186/s12864-024-10425-9 (PMC11137924; doi:10.1186/s12864-024-10425-9)
Supplement: Supplementary file 1 — Supplementary Material 1 [file 12864_2024_10425_MOESM1_ESM.doc]

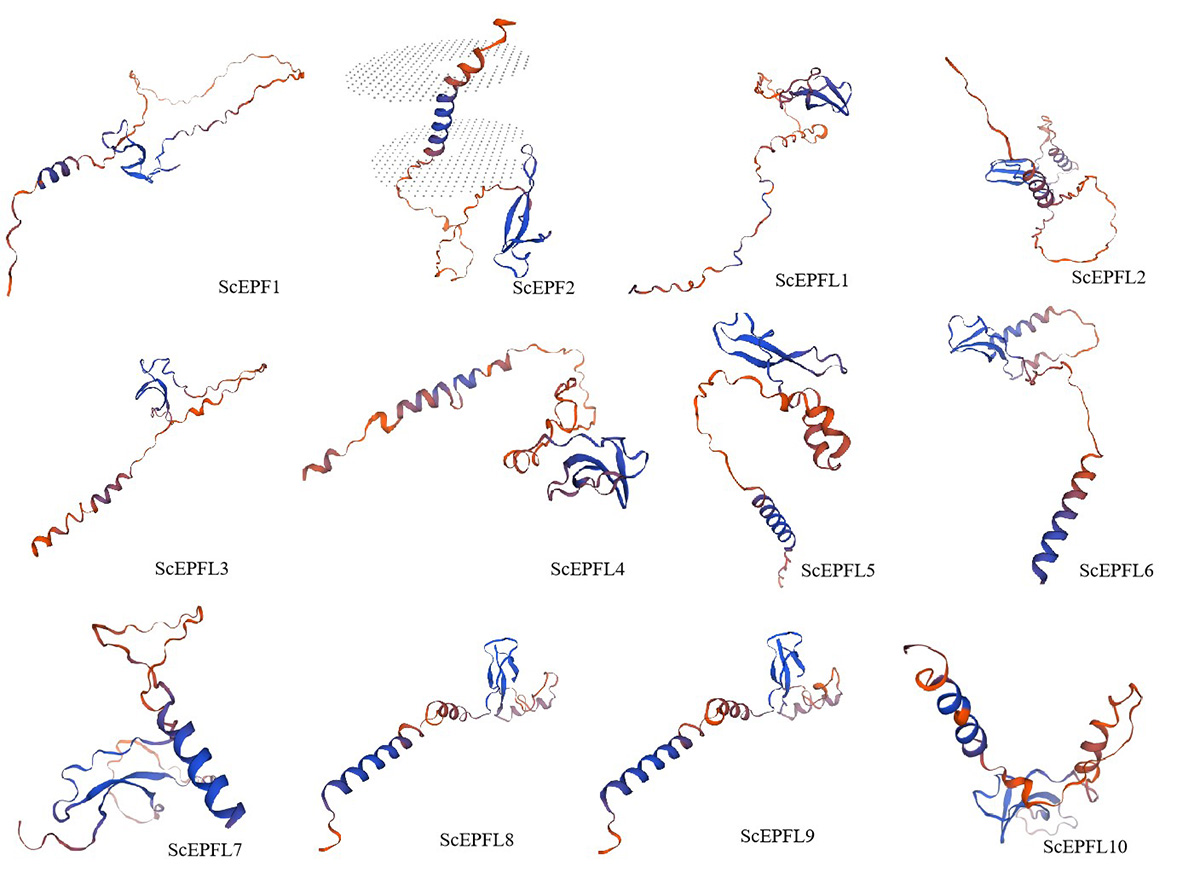
Fig. S1 Three-dimensional structures of EPF/EPFL proteins in rye


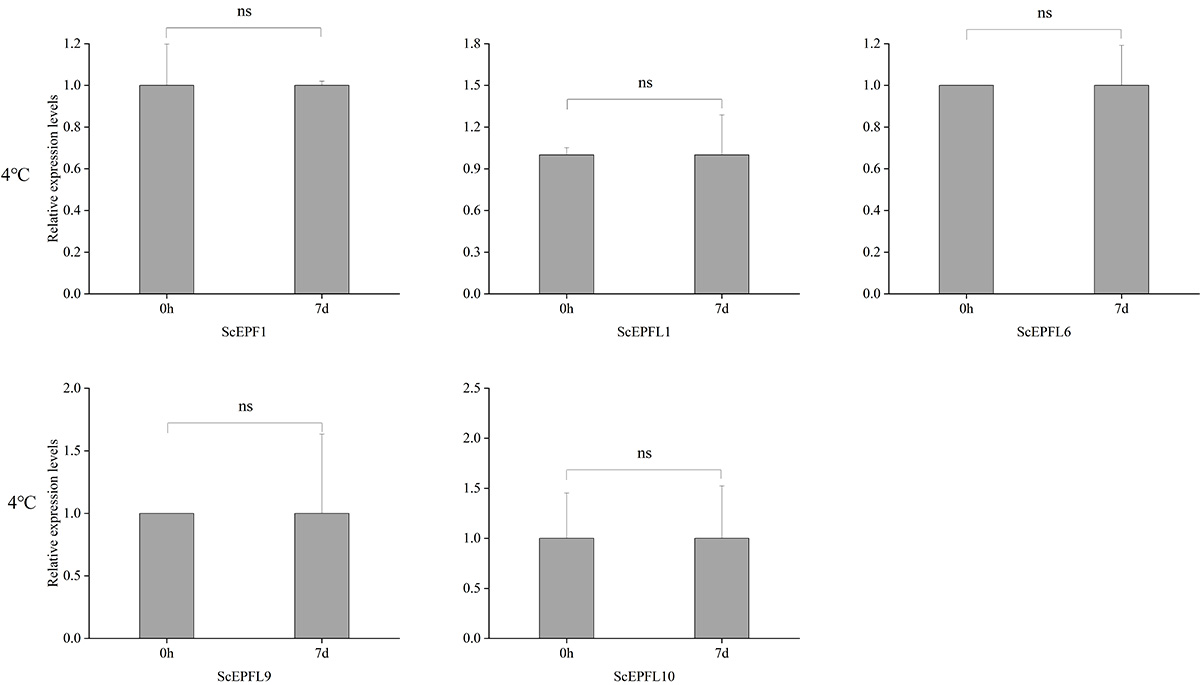


Fig. S2 Expression level of *ScEPF/EPFLs* under 4℃stress in rye
